# Clinical importance of systematic assessment and psychoeducation in specialised treatment of adolescents with severe functional somatic disorders

**DOI:** 10.1192/j.eurpsy.2022.386

**Published:** 2022-09-01

**Authors:** K. Kallesøe, A. Schröder, R. Wicksell, C. Rask

**Affiliations:** 1 Psychiatry, Aarhus University Hospital, Research Unit, Department Of Child And Adolescent Psychiatry, Aarhus N, Denmark; 2 Aarhus University Hospital, Research Clinic For Functional Disorders And Psychosomatics, Aarhus C, Denmark; 3 Karolinska Institutet, Department Of Clinical Neuroscience, Solna, Sweden; 4 Aarhus University, Department Of Clinical Medicine, Aarhus C, Denmark

**Keywords:** functional somatic disorders, psychoeducation

## Abstract

**Introduction:**

Functional somatic disorders (FSD) characterized by persistent and disabling physical symptoms are common in youth. Diagnostic uncertainty and insufficient illness explanations are proposed as perpetuating factors for FSD and may furthermore serve as barriers for treatment engagement.

**Objectives:**

The present study is part of a larger randomized trial and aimed at evaluating the impact of systematic assessment and psychoeducation on various clinical outcomes for adolescents suffering from severe FSD.

**Methods:**

Ninety-one adolescents (15-19 years) with severe FSD of at least 1 year’s duration were included in the randomized trial AHEAD (Acceptance and Commitment Therapy for Health in Adolescents). All participants received a thorough assessment (approximately 4 hrs.) and a subsequent psychiatric consultation (1.5 hrs) focusing on further psychoeducation and health promoting strategies. Clinical outcomes included self-reported physical health (SF-36), symptom severity, illness perception, illness related behaviour and psychological flexibility. Questionnaires were distributed at baseline (before assessment) and 2 months after randomisation. Data were analysed using simple t-tests.

**Results:**

Assessment and psychiatric consultation were not associated with a clinically relevant improvement of physical health, mean difference 0.23 95% CI [-0.95;1.41] p=0.701. However, a considerate decline was seen on symptom severity (p=0.017), illness worry (p<0.001) and negative illness perceptions (p<0.001). Furthermore, a decline was seen in limiting illness behaviour (p=0.002) and psychological inflexibility (p=0.001).

**Conclusions:**

The results underpin the importance and the potential positive implications of thorough assessment and psychoeducation. Hence, these elements may be in their own right in the systematic and specialised treatment of adolescents with severe FSD.

**Disclosure:**

No significant relationships.

